# Antithrombin III concentrate may contribute to sepsis in nonovert disseminated intravascular coagulation

**DOI:** 10.1186/cc12938

**Published:** 2013-11-05

**Authors:** Noriko Saito, Mari Yokota, Ahmin Kim, Tomoyuki Harada, Munekazu Takeda, Mizuho Namiki, Arino Yaguchi

**Affiliations:** 1 Department of Critical Care and Emergency Medicine, Tokyo Women's Medical University, Tokyo, Japan

## Background

Antithrombin III (AT III) has been known to contribute to anti-inflammatory response as well as its anticoagulation. Our previous study showed AT III deficiency happened in the early stage of sepsis with no relation to disseminated intravascular coagulation (DIC) status. Whether AT III concentrate is a beneficial therapy or not for septic patients is still a controversial issue. Our hypothesis is that AT III concentrate may have efficacy as an anti-inflammatory for sepsis.

## Materials and methods

From January 2009 to June 2013, adult septic patients with nonovert DIC whom were given AT III concentrate in our medico-surgical ICU were included in this study. DIC scoring was used with the definition of the International Society on Thrombosis and Haemostasis (ISTH). AT III concentrate was administered 30 to 60 U/kg intravenously every 24 hours for 3 days in the patients. Between before and after the AT III concentrate therapy, WBC (/mm^3^), CRP (mg/dl), platelet (×10^4^/μl), PT (seconds), fibrinogen (mg/dl), FDP (μg/ml), SOFA score and DIC score by ISTH were compared. Values are expressed as mean ± SD. Data were analyzed by Wilcoxon signed-rank test. *P *< 0.05 was considered significant.

## Results

There were 157 patients (100 men, 57 women; age range 19 to 96 years (mean 70.0 ± 16.0)), and the 28-day mortality rate was 25.5% and APACHE II score was 17.2 ± 8.3. WBC, CRP, PT, and SOFA score were significantly improved after AT III concentrate therapy (13,411 ± 8,794 vs. 11,798 ± 6,562, *P *= 0.0007, 17.1 ± 11.5 vs. 13.9 ± 7.0, *P *= 0.0001, 16.5 ± 10.9 vs. 15.2 ± 5.3, *P *= 0.002, and 8.6 ± 3.6 vs. 7.7 ± 4.5, *P *= 0.005, respectively), although platelet was significantly decreased (15.8 ± 11.3 vs. 13.7 ± 11.3, *P *< 0.00013). There were no significant differences in fibrinogen, FDP and DIC score (464.7 ± 235 vs. 437.6 ± 185.4, *P *= 0.10, 25.1 ± 36.9 vs. 25.6 ± 36.2, *P *= 0.85, 2.0 ± 1.5 versus 2.3 ± 1.7, *P *= 0.06, respectively). One week after the therapy, SOFA score was significantly improved, while the DIC score did not change compared with before the therapy (6.1 ± 4.7, *P *< 0.0001 and 2.3 ± 1.7, *P *= 0.98).

## Conclusions

In the patients with septic nonovert DIC, WBC, CRP and SOFA score were immediately improved after the AT III concentrate therapy, while fibrinogen, FDP and DIC score did not change. AT III concentrate may also contribute to anti-inflammatory without DIC status.

